# Quality of infant care in primary health services in Southern and Northeastern Brazil

**DOI:** 10.11606/S1518-8787.2018052000186

**Published:** 2018-01-29

**Authors:** Andréia Soprani dos Santos, Suele Manjourany Silva Duro, Nágela Valadão Cade, Luiz Augusto Facchini, Elaine Tomasi

**Affiliations:** I Universidade Federal do Espírito Santo. Centro Universitário Norte do Espírito Santo. Departamento de Ciências da Saúde. São Mateus, ES, Brasil; II Universidade Federal de Pelotas. Faculdade de Enfermagem. Pelotas, RS, Brasil; III Universidade Federal do Espírito Santo. Centro de Ciências da Saúde. Departamento de Enfermagem. Vitória, ES, Brasil; IV Universidade Federal de Pelotas. Departamento de Medicina Social. Pelotas, RS, Brasil

**Keywords:** Infant, Child Health Services, Quality of Health Care, Primary Health Care, Unified Health System, Family Health Strategy

## Abstract

**OBJECTIVE:**

To assess the quality of the health care provided to children aged under one year old performed by primary health services in the South and Northeast regions of Brazil.

**METHODS:**

This is a cross-sectional, population-based study carried out in 2010 with 7,915 children aged from one to four years, whose homes are located in the areas of health service coverage. We described the prevalence of procedures and guidelines, such as weight and height measurement, vaccination, newborn blood spot screening, evaluation of umbilical cord, instruction on breastfeeding and introduction of new food, and their respective 95% confidence intervals. The differences were analyzed using the chi-square test of heterogeneity and linear trend. We considered the main outcome of high-quality infant care if the child had received all recommended procedures and guidelines in the first year of life. For this analysis, we used the Poisson regression considering hierarchical model.

**RESULTS:**

There was low prevalence for the instruction on breastfeeding in the first week of life (58.8%, 95%CI 57.5–60.0) and on the introduction of new food in the fourth month care. The prevalence of high-quality in childcare was 42.0% (95%CI 40.5–43.5). The adjusted analysis according to hierarchical model indicated greater probability of this outcome in the Northeast region (PR = 1.17, 95%CI 1.09–1.26), in smaller municipalities (PR = 1.17, 95%CI 1.03–1.33), and in municipalities with 50,000 and 99,000 inhabitants (PR = 1.20, 95%CI 1.09–1.34).

**CONCLUSIONS:**

The Northeast region has higher-quality infant care services, which can be explained by the consolidation of the Family Health Strategy in that region.

## INTRODUCTION

The monitoring of child development and growth is a primary activity of health professionals, which is a standard practice regarding primary health care for this age group. Thus, childcare service from basic actions^23^ has a special position guaranteed in the healthcare policy agenda^25^.

The first years of a child are vital because they are a period of great vulnerability and adaptation to life conditions^3^; thus, they require continuous monitoring for a consistent promotion of health and prevention of diseases^23^. In the context of primary services, the Ministry of Health (MOH) recommends seven routine visits in the first year of life, distributed over the first week and the first, second, fourth, sixth, ninth, and twelfth months^20^. These services prioritize home visits in the early days of birth, instruction on breastfeeding, and control of vaccine-preventable diseases and childhood prevalent diseases^10^.

Infant care services comprise identification and instruction of parents about exclusive breastfeeding and performance of newborn blood spot screening test, analysis and report on weight and height, vaccination, and assessment of danger signs and possible vulnerabilities^20^. These actions, beyond their individual benefits, are able to generate health indicators that detail the profile of the services. Among those indicators, vaccination coverage, which shows the total percentage of children immunized, reflects the result of that service in each territory^4^.

Although care protocols for children are well established, there is still evidence of questionable service performance and variation in the quality^5^ ^,^ ^8^ ^,^ ^24^ because of region particularities^11^ and difficulties in implementing health promotion actions^12^ ^,^ ^14^ ^,^ ^24^. In that sense, the assessment of services becomes relevant to the organization and management of healthcare.

In Brazil, when considering the socioeconomic and demographic differences between the South and Northeast regions, the latter stands out for its high infant mortality and poverty rates, if compared to the South^22^. Since high quality care reflects on decreasing infant mortality^25^, we expected to find lower quality childcare in the Northeast region. Thus, this study aimed to assess the quality of the health care provided to children aged under one year old performed by primary health services in the South and Northeast regions of Brazil.

## METHODS

This cross-sectional, population-based study was conducted in the Northeast and South regions of Brazil, in areas covered by traditional primary health units (PHU) and Family Health Strategy units (FHS), randomly selected from urban census tracts. We identified in these areas the households with children aged under seven years old and their families. This study is part of the research “Health status, use of services, and quality of care for children and families in the South and Northeast regions of Brazil,” carried out from August to October 2010.

The municipalities were randomly selected among those covered between 30% and 70% by the Family Health Strategy, and they were stratified into four population scales: 10,000–29,999 inhabitants, 30,000–49,999 inhabitants, 50,000–99,999, and 100,000–999,999. We also randomized the urban PHUs from each random municipality using lists of address identification, care model, and census tracts in their area. For each PHU sampled, two census tracts were chosen: one that included health services and another that was contiguous.

In each sector, for 27 children, the starting point for the location of the households was randomly selected, always systematically jumping five households from the starting point. In each household, all children aged under seven years old were eligible for the study. For this analysis, the sample was restricted to children aged between one and four years, in order to know the full exposure to infant care at the end of the first year and to minimize possible recall bias by the mothers of older children.

The power of the sample was calculated afterwards, considering 42%^5^ for high quality prevalence of infant care services, that is if the child had received all the procedures recommended for the first year of life, margin for error of 1.7 percentage points, and a design effect of 2.0, adding up to 6,456 children. To verify the association between high quality childcare and household area, we used a significance level of 5%, power of 95%, exposed and unexposed rate of 1.0, prevalence of outcome among those unexposed of 40%, and prevalence rate of 1.13, amounting to 4,674 children. In addition to this number, we added 10% for losses or refusals and 30% for the control of confounding factors, resulting in 6,545 children. In total, 7,915 children aged from one to four years integrated the sample.

From the procedures performed – heel prick test, evaluation of the umbilical cord, vaccination, measurement, and instruction on weight and height, breastfeeding, and introduction of new foods –, a variable was constructed with the sum of the positive responses to all items in each of the seven of the services recommended in the first year of life.

Subsequently, a high-quality childbirth outcome variable was constructed if the child had received all the procedures and guidelines recommended in the first year of life.

The independent variables were geographic location: region (South, Northeast), size of the city in thousands of inhabitants (10–29, 30–49, 50–99, and 100–999), care model (family healthcare, traditional care); family socioeconomic status: economic classification of the Brazilian Association of Research Companies (ABEP – A/B, C, D/E), household income *per capita* in quartiles of minimum wages (up to 0.237, 0.238–0.431, 0.432–0.823, 0.824 or more), *Bolsa Família*– a Brazilian social welfare program – (yes, no); maternal demographic and social condition: age in years (up to 19, 20–29, 30–39, 40 or more), self-reported race (white, brown, black), school education in years (up to 4, 5–8, 9 or more), presence of spouse/partner (yes, no), total number of live births (1, 2, 3, or more); child status: total number of prenatal appointments (up to 5, 6 or more), sex (male, female), age in years (1, 2, 3, 4), race reported by the mother (white, brown, black).

Data collection was carried out by trained interviewers with the aid of a personal digital assistant (PDA). We used an individual questionnaire containing information on children and their mothers, as well as a socioeconomic questionnaire with information about the household and family. Interviews took place in the households and were answered by the biological mother or, in her absence, by a responsible guardian. For quality control, 8% of the interviews were repeated, when the sample answered again four questions about the child and family. Kappa statistics showed rates between 0.6 and 0.9, understood as good and excellent concordance, respectively^16^.

We calculated the prevalence and respective 95% confidence intervals (95%CI) for all procedures and instructions in the seven childcare appointments. We performed a bivariate analysis calculating the prevalence and associations with chi-square test of heterogeneity and linear trend. Poisson regression was used for the crude and adjusted analysis of the primary outcome, with robust adjustment of variance and prevalence ratios (PR), as well as 95%CI estimated by Wald test for heterogeneity and linear trend. The adjusted analysis was guided by hierarchical model with backward elimination. The input of variables happened by levels, considering the value p < 0.20, identified in the crude analysis. We included the geographic location of the variables in the distal level; the socioeconomic variables in the second one; and those related to maternal demographic and social characteristics, followed by the addition of proximal variables related to the child characteristics in the third level. At each level, the variables were adjusted among themselves and to higher levels. For all analysis, a 5% significance level was adopted. All data analyses were conducted using Stata 13.0.

This research was approved by the Ethics Committee of the Faculdade de Medicina of the Universidade Federal de Pelotas, registered under document number 133/09 in December 21, 2009. The informed consent was signed by all interviewees.

## RESULTS

In total, this sample consisted of 7,915 children aged one to four years, with predominance of residents in the Northeast region (51.0%), in municipalities with more than 100,000 inhabitants (59.7%), and in areas covered by FHS (81.8%). Most children belonged to families from economic class C (53.0%), half had an average monthly income *per capita* of less than 0.43 minimum wage (minimum wage value at the time was R$358.35, equivalent to US$203.72), and 35.1% were part of the *Bolsa Família* Program. More than half of the mothers were 20–29 years old (52.7%), 46.7% self-reported as white, 54.1% self-reported having nine or more years of school education, 78.8% lived with a spouse/partner, and 43.8% had only one child born alive. Regarding children, 85.7% were monitored in prenatal care with six or more visits, 52.0% were male, and 52.7% were white. The age factor was distributed equally among the four chosen years (Table 1).

**Table 1:** Description of the sample according to demographic, socioeconomic, maternal, and child variables. Southern and Northeastern Brazil, 2010.

Variable		n	%
Region (n = 7,915)			
	Northeast	4,041	51.0
	South	3,874	49.0
Municipality size in thousands of inhabitants (n = 7,915)			
	10–29	621	7.8
	30–49	1,565	19.8
	50–99	1,004	12.7
	100–999	4,725	59.7
Care model (n = 7,915)			
	Family Healh Strategy	6,478	81.8
	Traditional care	1,437	18.2
Economic classification (n = 7,455)			
	A or B	1,575	21.1
	C	3,951	53.0
	D or E	1,929	25.9
Income *per capita* in quartiles in minimum wage (n = 7.806)			
	≤ 0.237	1,767	24.9
	0.238–0.431	1,771	25.0
	0.432–0.823	1,783	25.2
	≥ 0.824	1,765	24.9
Social welfare program *Bolsa Família* (n = 7,777)			
	Yes	2,728	35.1
	No	5,049	64.9
Maternal age in years (n = 5,883)			
	≤ 19	469	8.0
	20–29	3,098	52.7
	30–39	1,875	31.8
	≥ 40	441	7.5
Maternal race (n = 5,861)			
	White	2,739	46.7
	Brown	998	17.0
	Black	2,124	36.3
Maternal school education in years (n = 5,582)			
	≤ 4	643	11.5
	5–8	1,919	34.4
	≥ 9	3,020	54.1
Presence of spouse/partner (n = 5,880)			
	Yes	4,633	78.8
	No	1,247	21.2
Number of live births (n = 5,893)			
	1	2,578	43.8
	2	1,924	32.6
	≥ 3	1,391	23.6
Number of prenatal appointments (n = 7,102)			
	≤ 5	1,016	14.3
	≥ 6	6,086	85.7
Sex of the child (n = 7,915)			
	Male	4,114	52.0
	Female	3,801	48.0
Age of the child in years (n = 7,915)			
	1	2,070	26.2
	2	1,968	24.8
	3	1,943	24.6
	4	1,934	24.4
Race of the child (n = 7,825)			
	White	4,123	52.7
	Brown	1,576	20.1
	Black	2,126	27.2

On the first week of life, the recommended actions were offered to more than 90% of the children, apart from instruction on breastfeeding, which reached only over half the sample. Except for instruction on breastfeeding, all actions were more frequent in the Southern Brazil. Instruction on the introduction of new foods had prevalence under 75.0% until the fourth-month appointment, increasing its results in the sixth-month appointment to 92.5% (95%CI 91.8–93.2). The prevalence related to the other actions was approximated in both regions. From the ninth-month of service onwards, information regarding instruction on breastfeeding and introduction of new foods were collected simultaneously, especially in the Northeast region (Table 2).

**Table 2:** Prevalence of procedures and health guidelines developed in infant care services in the first year of life, according to region. Southern and Northeastern Brazil, 2010.

Variable		Region (n = 7,915)		Total
		South (n = 3,874)	Northeast (n = 4,041)	
		% (95%CI)	% (95%CI)	% (95%CI)
1st week	Weight measurement	98.7 (98.2–99.0)	97.1 (96.4–97.6)	97.9 (97.5–98.3)
	Height measurement	98.5 (98.0–98.8)	96.0 (95.3–96.7)	97.3 (97.0–97.7)
	Instruction on breastfeeding	53.5 (51.8–55.2)	64.6 (62.9–66.3)	58.8 (57.5–60.0)
	Blood spot screening test	99.0 (98.6–99.3)	94.0 (93.1–94.8)	96.7 (96.2–97.1)
	Evaluation of the umbilical cord	93.3 (92.4–94.1)	87.4 (86.2–88.5)	90.6 (89.8–91.3)
1 month	Weight	99.7 (99.5–99.9)	99.5 (99.1–99,7)	99.6 (99.4–99.8)
	Weight report	97.5 (96.9–98.0)	97.6 (97.0–98,1)	97.5 (97.1–97.9)
	Height	99.2 (98.9–99.5)	97.0 (98.9–99,5)	98.2 (97.9–98.5)
	Height report	97.4 (96.8–97.9)	98.2 (97.6–98,6)	97.8 (97.4–98.1)
	Vaccination	98.8 (98.4–99.1)	98.7 (98.2–99,0)	98.8 (98.5–99.0)
	Instruction on breastfeeding	91.8 (90.8–92.7)	93.7 (92.8–94,5)	92.7 (92.0–93.3)
	Introduction of new foods	59.9 (58.3–61.6)	66.1 (64.4–67,9)	62.8 (61.6–64.0)
2 months	Weight measurement	99.6 (99.4–99.8)	99.3 (98.9–99.6)	99.5 (99.3–99.6)
	Weight report	97.5 (96.9–98.0)	97.9 (97.3–98.4)	97.7 (97.3–98.0)
	Height measurement	99.0 (98.7–99.3)	96.9 (96.3–97.5)	98.1 (97.7–98.4)
	Height report	97.3 (96.7–97.8)	98.5 (97.9–98.9)	97.8 (97.4–98.2)
	Vaccination	98.8 (98.4–99.1)	98.8 (98.4–99.2)	98.8 (98.5–99.1)
	Instruction on breastfeeding	92.2 (91.3–93.1)	95.7 (94.9–96.4)	93.8 (93.2–94.4)
	Introduction of new foods	62.0 (60.3–63.6)	70.1 (68.4–71.7)	65.7 (64.5–66.9)
4 months	Weight measurement	99.6 (99.3–99.8)	99.5 (99.1–99.7)	99.5 (99.3–99.7)
	Weight report	97.3 (96.7–97.8)	98.0 (97.4–98.4)	97.6 (97.2–98.0)
	Height measurement	98.9 (98.4–99.2)	97.3 (96.6–97.8)	98.1 (97.8–98.5)
	Height report	97.4 (96.8–97.9)	98.3 (97.7–98.7)	97.8 (97.4–98.2)
	Vaccination	98.7 (98.3–99.0)	99.0 (98.6–99.3)	98.9 (98.6–99.1)
	Instruction on breastfeeding	92.7 (91.8–93.6)	95.5 (94.7–96.2)	94.0 (93.4–94.6)
	Introduction of new foods	70.8 (69.2–72.3)	74.4 (72.8–76.0)	72.5 (71.3–73.6)
6 months	Weight measurement	99.6 (99.3–99.8)	99.6 (99.3–99.8)	99.6 (99.4–99.7)
	Weight report	97.6 (97.0–98.0)	98.0 (97.4–98.4)	97.8 (97.4–98.1)
	Height measurement	98.8 (98.3–99.1)	97.1 (96.4–97.7)	98.0 (97.6–98.3)
	Height report	97.5 (97.0–98.0)	98.4 (97.9–98.8)	97.9 (97.5–98.3)
	Vaccination	99.1 (98.8–99.4)	98.7 (98.2–991)	99.0 (98.7–99.2)
	Instruction on breastfeeding	92.9 (92.0–93.7)	95.5 (94.6–96.2)	94.1 (93.5–94.7)
	Introduction of new foods	91.4 (90.4–92.2)	93.8 (92.8–94.6)	92.5 (91.8–93.2)
9 months	Weight measurement	99.6 (99.3–99.8)	99.4 (99.0–99.6)	99.5 (99.3–99.7)
	Weight report	97.3 (97.0–98.1)	98.0 (97.4–98.5)	97.8 (97.4–98.2)
	Height measurement	99.0 (98.6–99.3)	97.1 (96.4–97.7)	98.2 (97.8–98.5)
	Height report	97.5 (96.9–98.0)	98.4 (97.8–98.8)	97.9 (97.5–98.3)
	Vaccination	99.0 (98.6–99.3)	98.7 (98.2–99.0)	98.9 (98.6–99.1)
	Breastfeeding and introduction of new foods	92.6 (91.7–93.4)	95.3 (94.4–96.0)	93.8 (93.2–94.4)
12 months	Weight measurement	99.4 (99.1–99.7)	99.5 (99.2–99.7)	99.5 (99.3–99.6)
	Weight report	97.5 (97.0–98.0)	98.3 (97.7–98.7)	97.9 (97.5–98.2)
	Height measurement	98.7 (98.3–99.1)	97.0 (96.2–97.5)	97.9 (97.5–98.3)
	Height report	97.7 (97.1–98.2)	98.5 (98.0–98.9)	98.1 (97.7–98.4)
	Vaccination	99.0 (98.6–99.3)	98.7 (98.2–99.0)	98.9 (98.6–99.1)
	Breastfeeding and introduction of new foods	92.4 (91.4–93.3)	95.6 (94.7–96.3)	93.9 (93.2-94.5)

Table 3 presents the prevalence of high quality childcare in each period. In the first week, 55.3% of the children had weight and height measurements, guidelines on breastfeeding, performance of the heel prick test, and evaluation of the umbilical cord.

**Table 3:** Prevalence of high quality outcome of child care in different periods, according to demographic, socioeconomic, maternal, and child variables. Southern and Northeastern Brazil, 2010.

Variable		1st week	1 month	2 months	4 months	6 months	9 months	12 months
		%	%	%	%	%	%	%
Region (n = 7,915)^a^		< 0.000	< 0.000	< 0.000	< 0.000	0.001	0.005	< 0.000
	Northeast	58.9	64.1	69.1	73.6	91.8	94.3	95.1
	South	52.1	57.7	60.4	69.1	89.3	92.4	92.5
Municipality size in thousands of inhabitants (n = 7,915)^b^		0.877	0.206	0.066	0.154	0.066	0.009	0.090
	10–29	55.7	66.0	71.0	76.7	89.7	93.0	94.1
	30–49	56.4	59.8	63.1	68.4	88.8	91.2	91.9
	50–99	52.1	60.7	66.5	77.0	91.3	93.3	93.7
	100–999	55.6	60.3	63.6	70.2	90.9	94.0	94.1
Care model (n = 7,915)^a^		0.234	0.501	0.807	0.590	0.104	0.669	0.046
	Family Healh Strategy	55.7	60.4	64.5	71.0	90.1	93.2	93.3
	Traditional care	53.8	61.5	64.1	71.8	91.7	93.6	94.9
Economic classification (n = 7,455)^b^		0.513	0.889	0.230	0.126	0.006	<0.000	0.005
	A or B	56.3	61.7	64.2	73.5	92.8	95.9	95.5
	C	54.8	59.5	62.9	69.8	89.7	92.4	92.7
	D or E	55.1	61.1	66.6	71.0	88.9	91.8	92.9
Income *per capita* in quartiles in minimum wage (n = 7.806)^b^		0.063	< 0.000	< 0.000	0.057	0.014	0.002	0.093
	≤ 0.237	56.4	64.7	69.5	73.3	88.5	91.3	92.7
	0.238–0.431	55.1	61.2	64.7	70.3	89.8	92.6	92.8
	0.432–0.823	55.2	58.0	62.2	69.7	91.4	93.8	93.7
	≥ 0.824	52.7	56.3	59.7	69.8	91.0	94.3	94.2
Social welfare program *Bolsa Família* (n = 7,777)^a^		0.149	0.081	0.010	0.685	0.195	0.023	0.365
	Yes	56.6	62.0	66.5	71.3	89.7	92.1	93.1
	No	54.7	59.7	63.1	70.8	90.7	93.8	93.8
Maternal age in years (n = 5,883)^b^		0.683	0.062	0.454	0.016	0.299	0.217	0.009
	≤ 19	49.7	57.1	62.8	65.8	88.8	89.7	90.7
	20–29	54.9	59.1	63.6	70.5	90.5	93.5	93.2
	30–39	53.1	60.2	63.4	71.3	90.7	93.9	94.5
	≥ 40	51.4	63.9	66.6	75.0	91.4	92.4	94.6
Maternal race (n = 5,861)^a^		0.040	0.008	0.001	0.459	0.084	0.114	0.220
	White	52.0	57.4	60.9	70.0	90.1	93.1	93.2
	Brown	57.0	61.5	67.2	72.2	89.2	91.9	92.7
	Black	54.5	62.0	66.0	71.1	91.8	94.2	94.4
Maternal school education in years (n = 5,582)^b^		0.711	0.785	0.478	0.219	0.016	0.036	0.121
≤ 4		54.7	59.1	62.9	71.0	91.3	94.1	94.1
5–8		52.2	61.1	65.0	71.3	90.3	93.1	93.1
≥ 9		54.1	58.1	63.1	67.0	87.7	91.5	92.5
Presence of spouse/partner (n = 5,880)^a^		0.230	0.227	0.044	0.383	0.668	0.662	0.310
Yes		53.2	59.2	63.0	70.5	90.4	93.2	93.3
No		55.4	61.4	66.6	72.0	90.9	93.6	94.3
Number of live births (n = 5,893)^b^		0.446	0.536	0.892	0.457	0.074	0.023	0.235
	1	53.3	58.5	64.0	70.7	90.8	93.7	93.7
	2	53.4	61.8	63.4	71.9	91.5	94.2	94.4
	≥ 3	54.8	58.9	63.8	69.0	88.4	91.2	92.2
Number of prenatal appointments (n = 7,102)^b^		0.526	0.038	0.025	0.781	0.016	< 0.000	< 0.000
	≤ 5	53.4	63.5	67.6	70.1	87.7	89.7	90.0
	≥ 6	54.6	59.4	63.2	70.6	90.6	93.6	93.9
Sex of the child (n = 7,915)^a^		0.319	0.783	0.970	0.229	0.301	0.524	0.080
	Male	55.9	60.5	64.4	71.8	90.1	93.1	93.1
	Female	54.6	60.8	64.4	70.4	90.9	93.5	94.2
Age of the child in years (n = 7,915)^b^		0.951	0.626	0.558	0.669	0.431	0.942	0.747
	1	55.2	60.7	65.4	70.7	90.8	93.3	93.9
	2	55.4	60.0	64.1	71.7	90.0	93.0	93.1
	3	55.6	60.3	63.4	70.4	91.3	93.9	94.4
	4	55.0	61.6	64.6	71.8	89.5	93.0	93.2
Race of the children (n = 7,825)^a^		0.001	0.005	0.001	0.361	0.506	0.132	0.246
	White	53.3	58.8	62.3	70.4	90.1	92.9	93.2
	Brown	56.5	62.4	66.5	72.5	91.1	93.1	94.0
	Black	58.5	63.2	67.4	71.6	90.9	94.5	94.5
Total		55.3	60.6	64.4	71.1	90.4	93.3	93.6

a Chi-square test of heterogeneity.

b Chi-square test of linear trend.

Quality of infant care ranged from 55.3% in the first week appointment to 93.6% in the twelfth-month appointment, with linear growth in the period. Quality of care was better shown in the Northeast region in all seven visits, performance that increased in both regions throughout the period. Municipality size above 100,000 inhabitants influenced the ninth-month visit. The same was observed in relation to the care model of health facilities in the twelfth-month visit. There were socioeconomic differences among classes (A/B, C, D/E) starting from the sixth-month appointment. Regarding income *per capita*, the differences were visible in the first-, second-, sixth-, and ninth-month appointments. Children who benefited from *Bolsa Família* also represented better quality performance in the second- and ninth-month appointments (Table 3).

We observed a higher prevalence of high quality childcare in mothers aged over 39 years in the fourth- and twelfth-month appointment; the greatest difference was found in the fourth-month appointment among mothers aged under 20 years (65.8%) and mothers aged over 39 years (75.0%). In both periods, the children of mothers aged up to 19 years had the lowest proportions of high quality (Table 3).

Children reported as brown and black by the mothers showed differences in the prevalence of quality in childcare in the first three appointments. A similar situation was identified regarding maternal education, whose contrast was observed in the sixth- and ninth-month appointment. Maternal characteristics related to the presence of spouse/partner and the number of live births showed insignificant results in association with the quality of childcare services. Mothers who had six or more prenatal appointments reported superior quality in three appointments. Regarding children, sex and age did not show significant differences. Race, on its turn, influenced the first three appointments (Table 3).

The prevalence of high quality childcare, considering all seven appointments in the first year of life, added up to 42.0% (95%CI 40.5–43.5). The adjusted analysis, according to the hierarchical model, showed upper prevalence of high quality in the Northeast region (PR = 1.17, 95%CI 1.09–1.26), in smaller municipalities (PR = 1.17, 95%CI 1.03–1.33), and in the group of 50,000–99,999 inhabitants (PR = 1.20, 95%CI 1.09–1.34). The other variables of the model, when submitted to adjustment in each level among themselves and in the levels among those with p < 0.05, did not show association with the quality of childcare services (Table 4).

**Table 4:** Crude and adjusted analysis of factors associated with high quality infant care services. Southern and Northeastern Brazil, 2010.

Variable		%	Crude analysis^a^		Adjusted analysis^a^	
			RP (95%CI)	p	RP (95%CI)	p
Region (n = 7,915)^b^				< 0.000		< 0.000
	Northeast	45.3	1.14 (1.06–1.22)		1.17 (1.09–1.26)	
	South	39.8	1.0		1.0	
Municipality size in thousands of inhabitants (n = 7,915)^b^				0.017		0.018
	10–29	47.3	1.17 (1.03–1.33)		1.17 (1.03–1.33)	
	30–49	42.4	1.05 (0.96–1.15)		1.03 (0.95–1.14)	
	50–99	46.2	1.14 (1.03–1.27)		1.20 (1.09–1.34)	
	100–999	40.4	1.0		1.0	
Care model (n = 7,915)^c^				0.306		
	Family Health Strategy	42.4	1.05 (0.95–1.14)			
	Traditional care	40.5	1.0			
Economic classification (n = 7,455)^c^				0.240		
	A or B	41.4	1.0			
	C	41.0	0.99 (0.91–1.08)			
	D or E	44.4	1.07 (0.97–1.19)			
Income *per capita* in quartiles in minimum wage (n = 7.806)^b^				0.001		0.100
	≤ 0.237	44.2	1.18 (1.06–1.32)		1.10 (0.96–1.26)	
	0.238–0.431	44.0	1.18 (1.06–1.31)		1.13 (1.00–1.27)	
	0.432–0.823	41.3	1.11 (0.99–1.23)		1.10 (0.99–1.22)	
	≥ 0.824	37.3	1.0		1.0	
Social welfare program *Bolsa Família* (n = 7,777)^b^				0.189		0.745
	Yes	43.4	1.05 (0.98–1.13)		0.98 (0.89–1.08)	
	No	41.3	1.0		1.0	
Maternal age in years (n = 5,883)^c^				0.738		
	≤ 19	37.1	1.0			
	20–29	41.0	1.11 (0.92–1.32)			
	30–39	39.6	1.07 (0.89–1.29)			
	≥ 40	38.2	1.03 (0.82–1.31)			
Maternal race (n = 5,861)^b^				0.148		0.961
	White	38.6	1.0		1.0	
	Brown	43.0	1.11 (0.99–1.25)		1.02 (0.88–1.18)	
	Black	41.1	1.07 (0.97–1.17)		0.99 (0.89–1.12)	
Maternal school education in years (n = 5,582)^c^				0.566		
	≤ 4	39.6	1.00 (0.87–1.16)			
	5–8	41.4	1.05 (0.96–1.15)			
	≥ 9	39.4	1.0			
Presence of spouse/partner (n = 5,880)^b^				0.105		0.138
	Yes	39.4	1.0		1.0	
	No	42.8	1.09 (0.98–1.20)		1.08 (0.97–1.21)	
Number of live births (n = 5,893)^c^				0.956		
	1	38.8	1.0			
	2	43.6	1.05 (0.96–1.16)			
	≥ 3	40.8	0.98 (0.88–1.10)			
Number of prenatal appointments (n = 7,102)^c^				0.258		
	≤ 5	39.5	1.0			
	≥ 6	41.6	0.94 (0.83–1.05)			
Sex of the child (n = 7,915)^b^				0.122		0.074
	Male	43.1	1.06 (0.99–1.13)		1.09 (0.99–1.19)	
	Female	40.8	1.0		1.0	
Age of the child in years (n = 7,915)^c^				0.964		
	1	41.4	1.00 (0.91–1.11)			
	2	42.6	1.03 (0.94–1.14)			
	3	42.9	1.04 (0.94–1.15)			
	4	41.2	1.0			
Race of the child (n = 7,825)^b^				< 0.000		0.128
	White	39.5	1.0		1.0	
	Brown	42.2	1.07 (0.97–1.18)		0.96 (0.82–1.12)	
	Black	47.2	1.20 (1.10–1.30)		1.11 (0.97–1.26)	
Total		42.0				

a Poisson Regression with robust variance.

b At each level, the variables were adjusted to each other and to higher levels, according to a hierarchical model.

c Variables not included in the adjusted model (value p > 0.20 in the crude analysis).

## DISCUSSION

The prevalence of procedures and health instructions received in infant care services indicated, in most cases, that PHU and FHS in the Northeast and South regions of Brazil have conducted the recommended health actions. The higher prevalence for measures of weight and height, greater than 97.0%, for all visits, suggests that health professionals have performed the anthropometric measures, which are important for the evaluation of child growth^23^. In a country where child obesity rates rise quickly, that practice reveals itself to be even more necessary and propitious for preventing childhood and adult chronic diseases^15^ ^,^ ^21^. Vaccination, on its turn, also stands out for presenting elevated prevalence, reflecting a well-structured and organized National Immunization Policy (NIP), which has been providing Brazilians with all types of vaccines. The NIP displays an efficient coverage, represented by its high rates even among low income populations^4^, which presupposes no obstacles to its access.

The low prevalence concerning instruction on breastfeeding carried out in the first-week appointment is alarming, as it is a critical period for the consolidation of breastfeeding, with information on baby sucking and feeding on demand^26^. All this information is essential to support the breastfeeding practice and to ensure proper weight gain to be evaluated in subsequent appointments. When that instruction is not considered, it deconstructs a preventive practice recommended over the years, which admittedly benefits the mother and baby^26^. Although this study has not investigated the reasons for such low prevalence, it represents a real drawback that seems to reside not in the absence of equipment but in the incompleteness of the service.

The professional responsible for infant care must guide and instruct the mothers and families of the children on the adoption of recommended behaviors^23^. The lack of professional training and understanding about the importance of appropriate monitoring of child growth may reflect gaps in service and result in failure to give instructions and guidelines to parents^6^ ^,^ ^7^ ^,^ ^12^.

In this study, the prevalence of instructions on the introduction of new foods met the standard recommended for feeding infants aged under six months^20^, being reduced in the first sessions, and followed by an increase in the sixth-month visit. From the first day of birth to the sixth month of the child's life, health professionals should not advise the ingestion of food other than breast milk^18^; after this period, it is necessary to instruct parents about the transition from exclusive breastfeeding to the beginning of ingestion of other foods^18^. It is noteworthy that this study did not set out to identify the duration of breastfeeding but the instruction provided by health professionals to parents in infant care services.

Brazilian studies have assessed the quality of childcare by completing health booklets, recording information on files, counting anthropometric measures, and performing procedures according to service protocols, as well as by national research. An overview of this situation is displayed by the minor prevalence of the completion of growth charts^1^ and health booklets^9^. The prevalence of appointments offered to children has been less than 60%^10^ ^,^ ^15^, reaching less than 30% for the first-week appointment of the newborn^5^ ^,^ ^15^. Blood spot screening test has been carried out in 70.8% of the children and the three doses of the tetravalent vaccine has reached 75.9%^15^. In general, we observed little favorable conditions for good quality of infant healthcare^1^ ^,^ ^5^ ^,^ ^7^ ^–^ ^11^ ^,^ ^17^ ^,^ ^19^.

When assessing the prevalence of high quality childcare throughout the seven sessions, in the first-week, first-, second- and fourth-month appointments, we observed that children were not entirely covered by the recommended health actions in the period. Reduced prevalence of instruction on breastfeeding and introduction of new foods may have affected this result.

It is also possible that health teams have failed to achieve certain basic procedures^12^. On the other hand, it was clear that children experienced nearly all health actions listed in the sixth-, ninth-, and twelfth-month appointments.

In all seven recommended visits, the Northeast region stood out for demonstrating the highest prevalence of quality in childcare, contrary to the study hypothesis, and showing adequate performance of health services in the Brazilian territory. In the adjusted analysis, the high quality infant care outcome was also higher in the Northeast (PR = 1.17, 95%CI 1.09–1.26). The explanation for this finding may be the fact that the Northeast region was the first to have the Program of Community Health Agents (PCHA) in the 1990s, and later the Family Health Program (FHP), currently named FHS^22^.

Over the years, the consolidation of the FHS program in the Northeast may have contributed to the current evidence of higher quality childcare services in that region. Possibly, health professionals of the FHS have more training, such as continuous education and residency courses in family healthcare, which qualify them to offer proper care and work in a multidisciplinary way, resulting in fulfillment of protocols and better performance in health services^2^. The expansion of the FHS program from 1999 to 2004 was more solid in the Northeast, in comparison to the South^13^. According to the 2008 National Household Survey, the proportion of residents enrolled in FHS units was higher in the Northeast (67.7%) rather than in the South (53.0%). According to the 2013 National Health Research, the Northeast region also had the highest percentage of residents in households registered in FHS units, 68.1%, in relation to other regions of Brazil; the South, for example, had 58.4%^17^.

The FHS contributed over the years to expand health services in small towns and outlying areas of major cities^22^, showing better results regarding the recommended actions carried out in the family health program when compared to traditional primary care^2^ ^,^ ^13^. These findings meet the results described herein, which revealed the highest quality of childcare among smaller municipalities (PR = 1.17, 95%CI 1.03–1.33) and among those with 50,000–99,999 inhabitants (PR = 1.20, 95%CI 1.09–1.34).

When analyzing the (unpublished) data of 220 health units in the South and Northeast regions, there were no differences in physical structure, inputs, human resources, and work process.

The adjusted analysis did not demonstrate the association of high quality infant care with variables linked to economy, mothers, and children. This result indicates that, in primary care, especially in infant care services, there seems to be no influence of these issues on the quality of infant healthcare, which strengthens health equity. This may be related to the expansion of the FHS throughout the country, which has particularly affected small municipalities, characterized by lower socioeconomic development^22^. According to Malta et al.^17^, in Brazil and in all regions, the FHS coverage has been higher in lower education areas, both regarding the proportion of registered individuals and home visits by a community health worker or team member. However, there is no denying that the existence of barriers, triggered by inadequate organization of healthcare services and by regional differences, interfere in the quality of care^14^.

It is important to underline that this study has limitations because of the research instrument, as it did not explore the reasons related to the low prevalence of some variables, such as instruction on breastfeeding, identified in the first-week appointment, and it does not address information about the neuropsychomotor development of the child. Another aspect to be observed is due to the sample procedure, since mothers of several children from the same family could get confused and switch information about their children. In order to minimize this problem, the interviewer was instructed to follow the individual script, according to the number of children in the residency. There is still the possibility of memory bias in the mothers’ interview. To avoid such risk, we collected only the information on appointments carried out with children aged under one year old.

This study gathered information using a household survey answered by the mothers about the care and instruction on infant care. These results reinforce the need to achieve complete and proper care services for children throughout their first year of life. Moreover, they point out the Northeast region as the highest in quality of infant care, possibly influenced by the consolidation of the FHS program in that territory.
